# Substance Use/Dependence in Psychiatric Emergency Setting Leading to Hospitalization: Predictors of Continuity of Care

**DOI:** 10.3390/ijerph19020760

**Published:** 2022-01-11

**Authors:** Angelo Giovanni Icro Maremmani, Mirella Aglietti, Guido Intaschi, Silvia Bacciardi

**Affiliations:** 1 Department of Psychiatry, North-Western Tuscany Region NHS Local Health Unit, Versilia Zone, 55049 Viareggio, Italy; silvia.bacciardi@uslnordovest.toscana.it; 2 Association for the Application of Neuroscientific Knowledge to Social Aims (AU-CNS), 55045 Lucca, Italy; 3 PISA-School of Clinical and Experimental Psychiatry, 56100 Pisa, Italy; 4 SERD (Drug Addiction Service), Department of Psychiatry, North-Western Tuscany Region NHS Local Health Unit, 55049 Viareggio, Italy; mirella.aglietti@uslnordovest.toscana.it (M.A.); guido.intaschi@uslnordovest.toscana.it (G.I.)

**Keywords:** psychiatric hospitalization, treatment adherence, continuity of care, substance use, emergency department, urinalyses, SERD, Italy

## Abstract

Background: Poor adherence to treatment is a common clinical problem in individuals affected by mental illness and substance use/dependence. In Italy, mental care is organized in a psychiatric service and addiction unit (SERD), characterized by dual independent assets of treatment. This difference, in the Emergency Room setting, leads to a risk of discontinuity of treatment in case of hospitalization. In this study we clinically characterized individuals who decided to attend hospital post-discharge appointments at SERD, in accordance with medical advice. Methods: This is a retrospective study, based on two years of discharged records of patients entering “Versilia Hospital” (Viareggio, Italy) emergency room, with urinalyses testing positive for substance use, and hospitalization after psychiatric consultation. The sample was divided according to the presence or absence of SERD consultation after discharge. Results: In the 2-year period of the present study, 1005 individuals were hospitalized. Considering the inclusion criterion of the study, the sample consisted of 264 individuals. Of these, 128 patients attended post-discharge appointments at SERD showing urinalyses positive to cocaine, opiates, and poly use; they were more frequently diagnosed as personality disorder and less frequently as bipolar disorder. The prediction was higher for patients that had already been treated at SERD, for patients who received SERD consultation during hospitalization, and for patients with positive urinalyses to cocaine and opiates at treatment entry. Conversely, patients who did not attend SERD consultation after discharge were affected by bipolar disorders. Limitations: Small sample size. Demographical data are limited to gender and age due to paucity of data in hospital information systems. SERD is located far from the hospital and is open only on weekdays; thus, it cannot ensure a consultation with all inpatients. Conclusions: Mental illness diagnosis, the set of substance use positivity at hospitalization, and having received SERD consultation during hospitalization appeared to have a critical role in promoting continuity of care. Moreover, to reduce the gap between the need and the provision of the treatment, a more effective personalized individual program of care should be implemented.

## 1. Introduction

In Italy, mental care is organized in psychiatric services and addiction units [1]. One of the critical points of the current Italian system is the presence of these dual independent assets of treatment, and this difference is especially evident in emergency rooms. Hospitalization is entrusted to Psychiatric Service Diagnosis and Treatment (SPDC), an inpatient treatment service located in the Hospital. Hospital emergency rooms provide 24/7 accessibility; individuals can access autonomously, by ambulance, or escorted by the police if needed. Addiction Units (SERD) are outpatient services organized to cope with the needs of individuals with drug addictions; they benefit from the presence of different professional expertise (doctors, psychologists, nurses, social workers, professional educators) to guarantee multidisciplinary interventions (e.g., outpatient visits or residential community programs) and work in collaboration with other local entities (services, self-help groups, multi-family communities, etc.). SERD are open daily during weekdays, and the access to these services is strictly voluntary. Psychiatrists working in these two settings belong to different equips: in accordance with this organization, subjects hospitalized for mental illness and substance use/dependence problems will be treated by different specialists leading to a risk of discontinuity of treatment and a more difficult management of patients’ needs. For this reason, there is a tight and continuous collaboration between psychiatrists working in SPDC and the ones working in SERD services, especially for patients affected by dual disorders.

In recent years, the expansion of substance use has led to an increase in voluntary and involuntary accesses to hospital emergency rooms. This became an issue to psychiatrists involved in emergency treatment settings who have to manage the acute phase of illness and then arrange a medium to long-term individual treatment program, placing significant demands on practitioners. As a matter of fact, high prevalence rates of coexisting mental health problems and substance misuse are present in both psychiatric treatment and addiction populations [2,3]. A retrospective study on clinical records from Centers for Medicare and Medicaid Services across different US states showed that individuals with severe mental illness (schizophrenia, bipolar disorder, or major depression) and a substance use disorder had higher odds of inpatient, emergency department, and hospital-based outpatient psychiatric treatment compared with those with severe mental illness alone [4]. The co-presence of mental illness and substance use/dependence is related with psychological, physical, and social complications, and these individuals are particularly difficult to engage with care. Often, awareness of illness can be reduced if not completely absent; because of this, treatment adherence can be negatively affected. Several studies focused on ways to improve treatment engagement. Previous studies show that among patients diagnosed with substance use/dependence in an emergency department setting, only 13% received follow-up at substance abuse services during the 14 days after their emergency department visit [5]. The post-discharge consultation appears to be critical; among patients discharged from residential treatment for substance use, the ones attending their initial outpatient appointment within 7 days of discharge evidenced better long-term outcomes relative to patients who did not, with respect to continuous abstinence [6]. Building a proper continuity of care would help reduce in-patient readmissions, considering that using substances precisely increases the odds of 30-day all-cause readmission (particularly opioid, cocaine, and amphetamine use) [7].

Predictably, voluntary admission for treatment, instead of enforced admission, may be helpful in increasing levels of motivation for treatment in substance users [8]. Different models of case management have proven effective in promoting engagement of people with substance use disorders in a variety of settings; particularly, “intensive case management for addiction” shows promising results [9]. Motivational interviewing is an evidence-based clinical method with strong empirical support for reducing problematic alcohol and drug use, but results suggest that the adherence in efficacy and effectiveness trials may be substantially different from that obtained in community treatment settings where adherence is likely to be far more heterogeneous [10]. A pilot randomized controlled trial looked at adjunctive psychosocial intervention following hospital discharge; participants randomized to enhanced assessment and monitoring showed a trend for increased treatment adherence over time [11]. Patient compliance or adherence is defined as the extent to which a patient’s behavior coincides with his or her prescribed medical or healthcare treatment [12]. In Italy, treatment providers encourage individuals during hospitalization to emphasize the importance of attending initial post-discharge appointments at SERD.

The aim of the present study is to point out clinical, toxicological variables and predictors of compliance in individuals who decided to attend post-discharge appointments at SERD, compared with their peers who did not attend.

## 2. Methods

### 2.1. Design of the Study

We conducted a retrospective study using 2018–2019 inpatient databases from the Department of Psychiatry informatics system where discharge records are stored. Demographical and clinical data were collected anonymously to preserve patient privacy. The present study included patients entering the emergency department of Versilia Hospital needing hospitalization after psychiatric consultation.

In Tuscany (Italy) the organization of mental health services is reflected at the national level [13]. “Versilia Hospital” (set in Viareggio, Tuscany) is organized with direct access to the emergency department that is next door to SPDC. SERD service, on the contrary, is located outside Versilia Hospital, about 10 km away.

As per internal SPDC protocol, inpatients who tested positive to urinalyses at treatment entry undergo a consultation with an addiction specialist (psychiatry) during hospitalization to enhance awareness of illnesses related to substance use/dependence and are encouraged to attend post-discharge appointments at SERD, promoting continuity of care.

### 2.2. Sample

We retrospectively considered all consecutive individuals hospitalized in the Psychiatric Department (SPDC) of “Versilia Hospital”, Lido di Camaiore, Italy, during a two-year period, 2018–2019. The inclusion criterion was

1. Presence of urinalyses positive to one or more substances.

The exclusion criteria were

1. Presence of urinalyses negative to any substance;
2. Absence of urinalyses test performed at hospitalization.

Considering the presence of SERD consultation after discharge from the hospital, it was possible to divide the sample into two subgroups: patients who completed SERD consultation after discharge and patients who did not. These two groups were then compared, at univariate and multivariate levels, with regard to clinical and toxicological variables.

### 2.3. Instruments

The following instruments were used to collect data on the variables to be studied.

#### 2.3.1. Demographic Data

We considered gender (males, females) and age at treatment entry. Because of the nature of the study, it was not possible to recall more demographic data on patients from data systems.

#### 2.3.2. Psychiatric Diagnosis

Mental health diagnoses were identified through the International Classification of Disease, Ninth Revision, Clinical Modification (ICD-9-CM) diagnosis codes included with each discharge record [14]. For the purpose of the present study, records that included a mental health ICD-9-CM code were recollected as follows:

- bipolar disorders (referring to affective psychosis);
- psychotic disorders (referring to paranoid states and schizophrenic psychosis with the exception of schizoaffective type);
- schizoaffective disorders (referring only to schizophrenic subtype of schizophrenic disorder);
- anxiety disorders (referring to neurotic disorders);
- adjustment disorders (referring to adjustment reaction);
- organic disorders (grouping together dementia and mental retardation);
- personality disorders.

Based on the diagnoses obtained, three mutually exclusive groups were created:

○ “Major psychiatry diagnosis” sums up major psychiatry diagnosis (bipolar disorders, psychotic disorders, schizoaffective disorders, anxiety disorders, adjustment disorders, organic disorders).
○ “Personality disorder” collects individuals with a personality disorder and without any other major psychiatric disorders.
○ “Substance users” highlights individuals without major psychiatric diagnoses and personality disorders.

#### 2.3.3. Used Substances

According to the urinalyses test utilized in the Psychiatric Department it was possible to check for 10 different substances: cocaine, methadone, opioids, benzodiazepines, amphetamines, MDMA (ecstasy), cannabinoids, ketamine, barbiturates, and alcohol.

#### 2.3.4. Data Analyses

We compared substance use, psychiatric diagnosis, length of hospitalization, “been previously treated at SERD”, and “perform SERD consultation during hospitalization” between patients who attended and did not attend post-discharge appointments at SERD service using chi-square and Student’s *t*-tests as appropriate (*p* < 0.05).

Multivariate logistic regression analyses were used to calculate odds ratios for the factors associated with “be patients who attended post-discharge appointments at SERD”, considering as predictors “been previously treated at SERD”, “SERD consultation during hospitalization”, major psychiatry diagnosis, personality disorder, and substance use positivity at treatment entry. All analyses were performed using SPSS 20.0 (IBM, Armonk, NY, USA).

## 3. Results

The number of individuals hospitalized in the 2-year period was 1005 (500 hospitalizations in 2018 and 505 in 2019). Considering the inclusion criterion of the study, the sample consisted of 265 individuals; 168 (63.4%) of them were male and 96 (36.6%) were female. The average age was 42.1 ± 12.7 (min 19, max 78).

One hundred twenty-eight patients completed SERD consultation after hospital discharge. Of them, 86 (64.8%) were male, and average age was 41.40 ± 11.1 (age ranged between 19 and 72).

One hundred thirty-seven did not receive SERD consultation after discharge. Of them 82 (59.9%) were male, and average age was 42.92 ± 14.6 (age ranged between 20 and 78). See Table 1 for descriptive analyses.

Table 2 shows that no differences were observed between patients who attended and did not attend post-discharge appointments at SERD with regard to age, sex, and length of hospitalization. In regard to substances used, patients who attended post-discharge appointments at SERD showed a high percentage of urinalyses positive to cocaine, opiates, and combined use of substances. Patients were less likely diagnosed with major psychiatric diagnoses, but they were more frequently diagnosed with personality disorder; among major psychiatric diagnosis, bipolar disorder showed a lower prevalence in the group of individuals who attended post-discharge appointments at SERD. Finally, patients that attended post-discharge appointments at SERD were more likely to have been previously treated at SERD (and therefore already known by SERD specialists), and they more frequently received SERD consultation during hospitalization.

Table 3 shows results of backward logistic regression including predictive factors identified in the “patients attended post-discharge appointments at SERD” group from the univariate analysis. On the basis of this analysis, the odds of attending post-discharge appointments at SERD were higher for patients that had already been treated at SERD, for patients who received SERD consultation during hospitalization, and for the ones with positive urinalyses to cocaine and opiates at treatment entry. Conversely, the odds were negative for patients affected by bipolar disorders.

## 4. Discussion

Patients who completed SERD consultation after discharge were less than half of the sample. Considering substances of abuse, these patients showed high percentages of urinalyses positive to cocaine, opiates (at a multivariate level), and combined use of substances (at univariate level). There is a lack of studies concerning the compliance to treatment in cocaine users after hospital discharge. In contrast with our results, a US study showed that lack of treatment request for cocaine problems was around 75% in cocaine users after emergency department visits [15]. Results related to opiates are not surprising considering that agonist opioid treatments are available and prescribed only at SERD services in Italy. Thus, to enroll in agonist opioid treatments patients have to access SERD units. As suggested by Raheemullah and colleagues, hospitalization could be a critical opportunity to move beyond treating the sequelae of opioid use disorder to treating the primary illness [16]. Opioid agonist treatment could be started or enhanced during hospitalization to stabilize patients from a clinical point of view, strengthening their insight and adherence to post-discharge programs at SERD services. The “combined use” result can be read according to a severity criterion, in which the higher number of used substances lead to a more complex clinical presentation and push to treatment request at SERD units. In line with this, a previous study highlighted that polysubstance users (notably, combined use of cocaine, alcohol, and marijuana) after hospital discharge appeared to be more motivated to seek help [17].

We found that completing SERD consultation after being discharged from the hospital was more frequent in patients already treated at SERD and for the ones who received a SERD consultation during hospitalization. Being already treated at SERD surely made patients aware of the availability of a treatment program after being discharged. Receiving a SERD consultation during hospitalization can even be seen as a form of brief psychosocial intervention that enhances insight of a patient on substance use/dependence, leading them to follow medical advice. A critical issue regarding inpatient consultation is that SERD is an outpatient clinic set outside Versilia Hospital, and it is not open 24/7. For this reason, it is possible that some individuals did not receive SERD consultation because the service was closed during the hospitalization stay.

According to mental illness diagnosis, patients who received SERD consultation after discharge were more frequently affected by antisocial personality disorder (borderline personality disorder nearly reaches statistical significance). According to our results, antisocial personality disorder and borderline personality disorder are the most common comorbid Axis II diagnoses among men and women with substance use disorders [18,19,20,21,22]. However, data about the proneness to treatment are unclear; it has been posited that certain personality types may be at increased risk of addiction and less likely to be motivated for treatment. [23]. On the contrary, and in line with our findings, antisocial personality disorder was more common in patients who were admitted voluntarily than in patients admitted via the courts [8].

Having a diagnosis of bipolar disorder was a predictor of not receiving SERD consultation after been discharged. It is surprising, especially because bipolar disorder has the highest rate of co-morbidity with substance use disorders; in fact, more than half of bipolar individuals were also diagnosed with an alcohol or drug use disorder. Extensive literature tells us about the prevalence rates of alcohol and/or drug use problems among these individuals [24,25,26], highlighting how these two disorders are highly debilitating and often comorbid conditions [27,28,29,30]. Epidemiological research suggests that rates of comorbid substance use disorders are higher in bipolar disorder than in any other psychiatric disorder [3,31,32,33,34,35]. The Epidemiologic Catchment Area study reported that over 60% of those with bipolar disorder had a comorbid substance use disorder, with 46% meeting criteria for alcohol use or dependence and 41% meeting criteria for drug use or dependence [2]. On the other side, individuals with a substance use diagnosis also have a significantly elevated risk of bipolar disorder, with rates estimated at 5–8 times greater than in the general population [2,3].

Studies focusing on comorbidity and patterns of substance use in hospitalized psychiatric patients show the greater prevalence of polysubstance use in individuals with mood disorders compared with other mental illness disorders [36]. Mood swings, impulsivity, and lack of control over one’s behaviors are critical during excitement episodes leading to risky behaviors such as substance use [11,37,38]. Particularly, risk-taking behavior was the only significant predictor of dropout in individuals with co-occurring bipolar disorder and alcohol dependence [39].

Because between 20% and 60% of bipolar disorder individuals seem to be non-adherent to medication, some authors developed adjunctive psychosocial interventions that target adherence in patients with bipolar disorder who are substance users. The intervention involves brief in-person sessions and follow-up phone contacts with the patient and a significant other/family members, to enhance the effects of existing treatments [40]. A northern Europe study shows that, irrespective of diagnosis (schizophrenia or schizoaffective disorder; bipolar disorder; depressive disorder), current outpatients had been clearly more adherent to preceding outpatient visits than current inpatients. Authors argue that hospital setting was the strongest clinical correlate of poor adherence in all diagnostic groups, and substance use disorder was another significant contributor to non-adherence in all three groups [41]. A previous study shows that rates of poor adherence to treatments are similar in schizoaffective disorder, bipolar type and bipolar disorder type I. Bipolar disorder type I patients with comorbid personality and substance use disorders are likely to be poorly adherent [42].

Surely the role of insight is critical. As pointed out by our results, receiving a previous consultation at SERD services and receiving a consultation during hospitalization are predictors of attending post-discharge appointments at SERD, highlighting the willingness to follow medical advice and continue treatment. Fostering insight during an inpatient stay may be an important part of reducing symptom severity and preventing patient relapse [43]. As was to be expected, patients applying voluntarily for treatment of substance use/dependence have higher motivation overall, including desire for help and treatment readiness [8]. The problem of lack of awareness of illness is well known in psychiatry leading to difficulty in managing individuals’ diseases. Relapse and craving are at the core of addiction, towards which individuals are syntonic. First phases of the toxicomanic process are distinguished by complete lack of insight and the thought of being able to freely dispose of one’s actions; only in time (even after years of illness) individuals begin to develop awareness of illness and, along with this, start to require treatment at addiction units [44,45]. In line with this, we could have speculated that individuals receiving SERD consultation after discharge would have been older, but the role of age, at least in our study, seems not to differentiate between the two groups.

Because of the existence of problems of adherence to treatment, the main goal of clinicians should be to bring patients closer to available treatments. Specific programs and psychoeducational interventions should be planned for this population to enhance insight and build compliance to treatment [11]. Generally, differences between psychiatric and substance use/dependence programs appeared to involve difficulties in developing treatments that were fully oriented toward the co-occurring diagnosis. To improve the provision of high-quality dual-focused care, it is recommended that planners use cross-system teams and applications of recently produced tools designed to increase a program’s ability to deliver integrated care to dually disordered individuals [46]. Nowadays, the Italian service system is not sufficiently developed and coordinated to serve patients with comorbid problems in the proper way. In order to reduce the gap between the need and the provision of the treatment, a more effective personalized individual program of care should be implemented even in light of an already applied effective model for chronic diseases known as “Shared Care” or “Mixed Care Model” [47,48].

## 5. Limitations

The first limitation to be addressed is the small sample size. Demographical data are limited to gender and age due to the nature of this study. The hospital information system does not allow one to find other data such as marital status, educational level, and work situation.

SERD consultation during hospitalization was performed in nearly half of inpatients. The reason for this was that SERD service is located far from the hospital and is open only on weekdays; thus, it cannot ensure a consultation with all inpatients. However, all inpatients that did not receive a proper consultation were invited by SPDC psychiatrists to attend post-discharge appointments at SERD, promoting continuity of care. New approaches related to E-mental health should be considered to increase patient involvement in the care process.

## 6. Conclusions

Mental illness diagnosis, the set of substance use positivity at treatment entry, and awareness of the role of SERD services (even with a SERD consultation during hospitalization stay) appear to play critical roles in promoting continuity of care. Clinicians should be aware of clinical data to provide a more precise and extensive treatment that would be fully oriented toward the co-occurring presence of mental illness and substance use/dependence. The main goal of clinicians should be to promote specific programs, enhancing insight and building compliance to treatment. Moreover, to reduce the gap between the need and the provision of the treatment, a more effective personalized individual program of care should be implemented, promoting continuity of care.

## Figures and Tables

**Table 1:** Descriptive analyses.

	N (%)
Gender (male)	168 (63.4)
Alcohol (urinalyses positive)	149 (56.2)
Cocaine (urinalyses positive)	85 (32.1)
Cannabis (urinalyses positive)	73 (27.5)
Opiates (urinalyses positive)	16 (6)
Benzodiazepines (urinalyses positive)	12 (4.5)
Others (urinalyses positive)	7 (2.6)
Combined use (more than 2 substances of abuse)	66 (24.9)
Combined use (more than 3 substances of abuse)	14 (5.3)
Agonist Opioid Treatment (ongoing)	37 (14)
Diagnosis	
Major Psychiatric Diagnosis	116 (43.8)
Substance users	80 (30.2)
Personality Disorders	69 (26)
Major psychiatric diagnosis	
Adjustment Disorder	31 (11.7)
Bipolar Disorder	27 (10.2)
Psychotic Disorder	26 (9.8)
Schizoaffective Disorder	13 (4.9)
Anxiety Disorder	9 (3.4)
Organic Disorder	6 (2.3)
No major psychiatric diagnosis	153 (57.7)
Personality disorders	
Borderline	31 (11.7)
Antisocial	23 (8.7)
Personality NAS	11 (4.2)
Histrionic	4 (1.5)
No personality disorder	196 (74)
Already treated at SERD	159 (60)
SERD consultation during hospitalization	134 (50.6)
SERD consultation after discharge	128 (48.5)
	M ± SD (min–max)
Age	42.1 ± 12.7 (19–78)
Length of Hospitalization (days)	5 ± 3.7 (1–28)

**Table 2:** Differences between patients who attended and did not attend hospital post-discharge appointments at SERD.

	SERD Consultation after Discharge	No SERD Consultation after Discharge		
	N = 128	N = 137		
	M ± SD	M ± SD	T	*p*
Age	41.40 ± 11.12	42.92 ± 14.06	−0.980	0.328
Length of Hospitalization	5.04 ± 3.42	5.04 ± 4.06	−0.010	0.992
	N (%)	N (%)	Chi^2^	*p*
Been previously treated at SERD	115 (89.8)	44 (32.1)	91.882	0.000
SERD consultation during hospitalization	83 (64.8)	51 (37.2)	20.191	0.000
Gender (male)	86 (67.2)	82 (59.9)	1.534	0.251
Psychiatric Diagnosis				
Major Psychiatric Diagnosis	46 (35.9) b	70 (51.1) a		
Substance users	41 (32) a	39 (28.5) a		
Personality Disorders	41 (32) a	28 (20.4) b	7.167	0.028
Bipolar Disorder (presence)	3 (2.3)	24 (17.5)	16.652	0.000
Psychotic Disorders (presence)	14 (10.9)	12 (8.8)	0.355	0.680
Schizoaffective Disorder (presence)	8 (6.3)	5 (3.6)	0.959	0.400
Anxiety Disorder (presence)	6 (4.7)	3 (2.2)	1.258	0.321
Adjustment Disorders (presence)	10 (7.8)	21 (15.3)	3.619	0.084
Organic Disorders (presence)	2 (1.6)	4 (2.9)	0.551	0.685
Borderline	20 (15.6)	11 (8)	3.696	0.059
Antisocial	16 (12.5)	7 (5.1)	4.560	0.048
Histrionic	2 (1.6)	2 (1.5)	0.005	1.000
Personality Disorders NAS	3 (2.3)	8 (5.8)	2.032	0.220
Alcohol (urinalyses positive)	66 (51.6)	83 (60.6)	2.188	0.173
Cocaine (urinalyses positive)	59 (46.1)	26 (19)	22.332	0.000
Cannabis (urinalyses positive)	32 (25)	41 (29.9)	0.805	0.410
Opiates (urinalyses positive)	14 (10.9)	2 (1.5)	10.478	0.001
Benzodiazepines (urinalyses positive)	3 (2.3)	9 (6.6)	2.733	0.086
Others (urinalyses positive)	5 (3.9)	2 (1.5)	1.540	0.268
Combined use (more than 2 substances of abuse)	40 (31.3)	26 (19)	5.328	0.023
Combined use (more than 3 substances of abuse)	13 (10.2)	1 (0.7)	11.751	0.001

Letters a and b denote differences for *p* < 0.05.

**Table 3:** Step-wise logistic regression analysis. Patients attended “post-discharge appointments at SERD” is the criterion. Variables showing statistical significance at univariate level are predictors.

Predictors	STEP	EXP (B)	Min	Max	*p*
Already treated at SERD	1	15.26	7.35	31.68	0.000
Cocaine (urinalyses positive)	2	3.19	1.58	6.45	0.001
Bipolar Disorder (presence)	3	0.13	0.03	0.53	0.005
SERD consultation during hospitalization	4	2.43	1.26	4.67	0.008
Opiates (urinalyses positive)	5	5.44	0.95	31.11	0.057

Statistics: chi square = 136.09, df = 5, *p* = 0.000.

## Data Availability

Authors can be contacted for information about dataset.
